# Exploring Roles of the Polysaccharide Capsule in Pathogenesis of Hypervirulent *Acinetobacter baumannii* Clinical Isolate Lac-4

**DOI:** 10.3390/antibiotics13010010

**Published:** 2023-12-20

**Authors:** Elisabet Bjånes, Truman Koh, Tariq Qayum, Raymond Zurich, Sinead McCabe, Kegan Hampel, Lisa Cartwright, Victor Nizet

**Affiliations:** 1 Division of Host-Microbe Systems and Therapeutics, Department of Pediatrics, University of California San Diego, La Jolla, CA 92093, USA; tkoh@ucsd.edu (T.K.); mtqayum@ucsd.edu (T.Q.); rhzurich@ucdavis.edu (R.Z.); sinead.mccabe.g4@kyowakirin.com (S.M.); kegan@inhibrx.com (K.H.); lcartwright@student.sdccd.edu (L.C.); 2 Skaggs School of Pharmacy and Pharmaceutical Sciences, University of California San Diego, La Jolla, CA 92093, USA

**Keywords:** *Acinetobacter baumannii*, bacterial infections, antibiotic resistance, pneumonia, sepsis, polysaccharide capsule, complement, neutrophils

## Abstract

The frequently multidrug-resistant bacterial pathogen *Acinetobacter baumannii* is a leading cause of nosocomial infections, including ventilator-associated pneumonia, such that the World Health Organization and US Centers for Disease Control and Prevention have declared it a top priority candidate for novel drug development. Nearly all clinical *A. baumannii* strains express a thick surface polysaccharide capsule that protects against desiccation, host defenses, and disinfectants. In this study, we investigated the contribution of the polysaccharide capsule to virulence caused by the *A. baumannii* clinical isolate Ab Lac-4, which is rare in its ability to cause pneumonia and disseminated sepsis in healthy mice. We assessed the role of the capsule in wildtype Lac-4 (WT) by generating a premature stop codon in *wza*, which codes for the polysaccharide export protein. The *wza#* mutant was hypersensitive to killing by complement, whole blood, and healthy human neutrophils compared to WT and a revertant mutant (*wza-Rev*). Furthermore, the *wza#* mutant was highly attenuated in murine sepsis and unable to disseminate from the lungs during pneumonia. This study reinforces the capsule as a key contributor to Ab Lac-4 hypervirulence.

## 1. Introduction

The Gram-negative opportunistic bacterial pathogen *Acinetobacter baumannii* is a frequently multidrug-resistant (MDR) and rapidly expanding cause of ventilator-associated pneumonia (VAP), bacteremia, urinary tract infections, and wound infections [1,2,3]. In certain regions, over 80% of *A. baumannii* clinical isolates exhibit MDR phenotypes [4,5]. In particular, carbapenem-resistant *A. baumannii* (CRAB) strains pose a formidable challenge given their resistance to nearly all antibiotic classes, including carbapenems, typically reserved as a last line of defense [4,5]. Given its high resistance levels and significant clinical impact, CRAB has been designated as the top priority for developing novel therapeutic interventions by the World Health Organization and the U.S. Centers for Disease Control and Prevention [6,7]. 

Like numerous other multidrug-resistant (MDR) pathogens, most *A. baumannii* clinical isolates express a dense polysaccharide capsule, recognized as a pivotal virulence factor in infections [8,9,10,11,12]. The genetic arrangement responsible for this capsule, referred to as the K locus, exhibits consistency in its export genes, succeeded by a highly variable complex sugar synthesis region, and concludes with conserved simple sugar synthesis genes [8]. The size of the K locus can vary, ranging from 20–35 kB, reflecting genetic diversity in the complex sugar synthesis region. This variability has led to the identification of over 100 unique K loci.

Polysaccharide capsules are believed to play a central role in *A. baumannii*’s repertoire of virulence determinants, acting as a protective barrier against host immune defenses, antimicrobial agents, other microbes, or challenging environments [1,9,13,14]. Hyperproduction of capsules has been associated with increased virulence in *A. baumannii* clinical isolates [15,16]. Despite the upregulation of capsule synthesis genes observed after exposure to certain antibiotics, it remains unclear whether this protective response is specific to particular *A. baumannii* strains and capsule types or if it represents a universal defense against antibiotic stress. 

*A. baumannii* is a human-adapted pathogen, and until recently, successful infection of mice required high inoculums or suppression of the murine immune system to mimic the disease presentation of pneumonia or sepsis in humans or to observe mortality [1]. The hypervirulent Ab Lac-4 strain, isolated from a Los Angeles County Hospital, induces sepsis and pneumonia in mice at lower doses than commonly studied strains such as AB5075 [17,18]. The whole genome sequence of Ab Lac-4 [19] reveals an unusually large capsule locus with several unique genes compared to AB5075. To understand the potential contribution of the polysaccharide capsule to the hypervirulence of Ab Lac-4, we disrupted the gene encoding capsular polysaccharide export protein (Wza) to generate a capsule-deficient mutant, *wza*#. Compared to the wildtype (WT) Ab Lac-4, the polysaccharide capsule provides protective advantages against antibiotics and components of the innate immune system in blood, including complement-mediated lysis and neutrophil opsonophagocytic killing. Moreover, the loss of the capsule also severely attenuates *wza#* in murine pneumonia and sepsis models compared to WT controls, limiting its ability to disseminate to systemic organs. These findings highlight the crucial role of the polysaccharide capsule in the virulence of *A. baumannii* clinical isolates, especially Ab Lac-4, a valuable strain for studying pathogenesis and evaluating therapeutic interventions in murine models of invasive infection.

## 2. Results

### 2.1. Generation of an A. baumannii Lac-4 Capsule-Deficient Mutant

**Figure 1:**
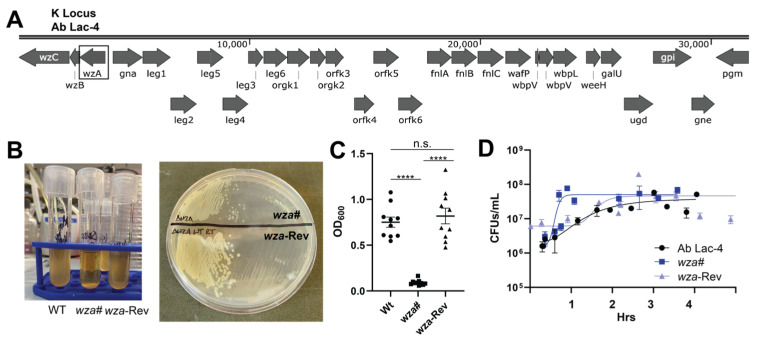
Gene deletion strategy: (**A**) Schematic depicting capsule locus (K Locus) for *Acinetobacter baumannii* Ab Lac-4. (**B**) Representative images showing the impact of capsule loss on buoyancy and growth on tryptic soy agar (TSA). Ab Lac-4 WT, *wza*#, and *wza*-Rev were grown to stationary phase and allowed to stand at RT for 3 h. (**C**) Turbidity (OD_600_) of the liquid cultures; three independent experiments pooled. n.s. = not significant, **** *p* < 0.0001. (**D**) Growth curves from Ab Lac-4, *wza*#, and *wza*-Rev grown in tryptic soy broth (THB). Three independent experiments were pooled.

To determine the contribution of the polysaccharide capsule in the hypervirulent strain Ab Lac-4 (hereafter designated wildtype, WT), we identified the K locus present in the published whole genome sequence of this strain (Figure 1A) [19]. Subsequently, we created a mutant in the gene encoding the capsular polysaccharide export protein, *wza* [20], utilizing a mutagenesis approach based on a two-selection process with the suicide vector pMo130-Apr (Figure S1A,B) [21]. The procedure involved generating single-crossover insertional mutants via apramycin selection, confirmed using PCR (Figure S1C), followed by double-crossover deletion mutants selected by serial passage in 20% sucrose. The resulting candidate mutant, named *wza*#, displayed reduced buoyancy and a paler, non-mucoid appearance compared to the WT strain. (Figure 1B,C). Surprisingly, the *wza* gene was still detected during PCR analysis (Figure S1D), prompting sequencing that revealed an interrupted *wza* gene with a premature stop codon (111Q replaced by a C → T substitution at base 331). A revertant strain, *wza*-Rev, was identified using serial passage, regaining WT buoyancy and mucoid phenotype. Both *wza*# and *wza*-Rev showed no growth defects in bacteriological media compared to parental WT *A. baumannii*, confirming that the capsule is not essential for in vitro growth (Figure 1D). 

### 2.2. Capsule Contribution to A. baumannii Lac-4 Antibiotic Susceptibility

Considering the potential of polysaccharide capsules to serve as a physical defense against antibiotics, we subjected *wza*# to testing with various antibiotics. The results revealed a slightly increased susceptibility of *wza*# to streptomycin and vancomycin compared to WT Ab Lac-4 (Table 1). These modest effects did not reach statistical significance and may reflect the complex and redundant repertoire of *A. baumannii* antibiotic resistance mechanisms, including multiple efflux pump systems.

### 2.3. A. baumannii Lac-4 Capsule Promotes Resistance to Human Innate Immune Factors

**Figure 2:**
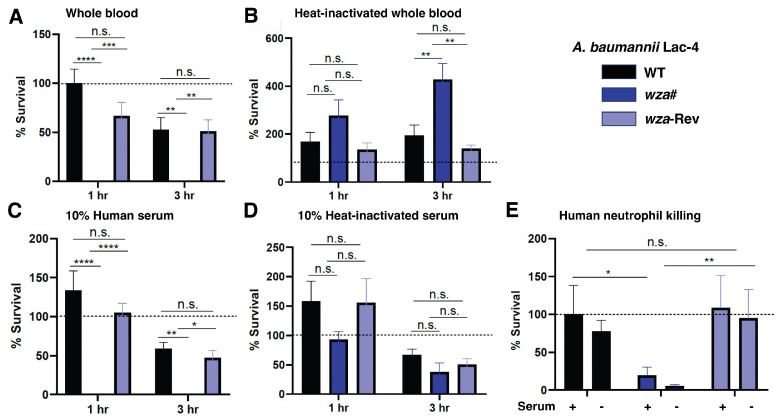
Loss of polysaccharide capsule significantly increases susceptibility to the innate immune system. Percent survival of Ab Lac-4, *wza*#, and *wza*-Rev in the presence of healthy human (**A**) whole blood, (**B**) heat-inactivated whole blood, (**C**) 10% active human serum, and (**D**) 10% heat-inactivated human serum for 1 or 3 h. (**E**) Percent survival of serum opsonized WT, *wza*#, and *wza*-Rev 3 h after incubation with healthy human neutrophils. The means of three independent experiments ± SEM are graphed. n.s. not significant, * *p* < 0.05, ** *p* < 0.01, *** *p* < 0.001, **** *p* < 0.0001.

In our subsequent investigation, we directed our focus toward comprehending the role of the Ab Lac-4 polysaccharide capsule in evading the human immune system. To achieve this, we incubated WT, *wza*#, and *wza*-Rev with whole blood from healthy human donors. Our finding showed the absence of the capsule significantly heightened the susceptibility of *wza*# to whole-blood killing (Figure 2A). This susceptibility was contingent on the presence of active complement components, as heat inactivation restored the survival of the capsule-deficient mutant (Figure 2B). Similarly, *wza*# exhibited high susceptibility to killing by 10% active human serum in a complement-dependent manner (Figure 2C,D). Finally, the neutrophil is a frontline innate immune cell that represents an early responder to the site of *A. baumannii* infection and is critical for infection control [22,23]. We discovered that neutrophils isolated from healthy human donors were markedly more effective at killing the *wza*# mutant compared to the parental WT or *wza*-Rev strains (Figure 2E). This underscores the capsule’s significance in defending against phagocytosis and provides confirmation of its crucial role in countering key clearance mechanisms of the human innate immune system.

### 2.4. Capsule Contributes to A. baumannii Lac-4 Hypervirulence in Murine Infection Models

**Figure 3:**
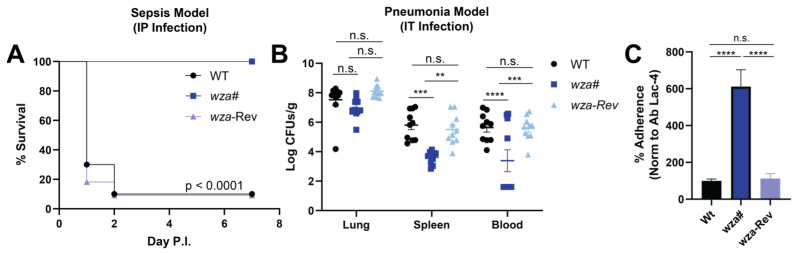
The capsule is required to cause lethal sepsis and systemic dissemination from the lungs during pneumonia. (**A**) Survival data from C57BL/6 (B6) mice infected intraperitoneally (IP) with 1 × 10^7^ CFUs WT, *wza*#, or *wza*-Rev in PBS. n = 10, two independent experiments pooled. (**B**) CFUs from lungs, spleens, and blood collected 12 h post-infection with 1 × 10^7^ CFUs WT, *wza*#, or *wza*-Rev intratracheally (IT). Mice were humanely euthanized according to IACUC protocols; organs were collected, homogenized, serially diluted in sterile PBS, and plated for CFUs on TSA. Blood was collected using cardiac puncture. n = 10, two independent experiments were pooled. (**C**) Percent adherence to A549s relative to WT. WT, *wza*#, and *wza*-Rev were incubated with A549 cells for 30 min. Cells were washed 5× with PBS, trypsinized with 0.05% trypsin, serially diluted in sterile PBS, and plated for CFUs. Means ± SEM of three independent experiments. n.s. not significant, ** *p* < 0.01, *** *p* < 0.001, **** *p* < 0.0001.

Despite *A. baumannii* being a human-adapted pathogen, the Ab Lac-4 strain demonstrates high virulence in mice, leading to disseminated sepsis [17]. However, the loss of the capsule significantly attenuated Ab Lac-4 in vivo. Mice challenged systemically with the *wza*# mutant strain all survived, whereas 90% of those infected with WT or *wza*-Rev strains succumbed within 48 h (Figure 3A). The Lac-4 strain is notable for its ability to disseminate from the lung to systemic organs after intratracheal infection [17], requiring 100- to 1000-fold lower inoculums than other A. baumannii strains. We explored whether the capsule contributes to Lac-4’s systemic dissemination during pneumonia. Twelve hour post-intratracheal challenge, comparable bacterial levels were recovered from the lungs of animals infected with each of the three strains. However, mice infected with the Lac-4 *wza*# mutant exhibited approximately 2-log10-fold fewer colony-forming units (CFU) of *A. baumannii* in the blood or spleen compared to those infected with WT or *wza*-Rev (Figure 3B). These findings are consistent with prior studies showing other acapsular mutant strains of *A. baumannii* are attenuated in vivo [8,11].

### 2.5. Capsule Expression Reduces A. baumannii Lac-4 Lung Epithelial Cell Adherence

The presented data highlight the indispensability of the polysaccharide capsule for *A. baumannii* Lac-4 dissemination from the lungs in pneumonia and proliferation in the bloodstream. However, the maintenance of similar CFU counts within the lungs suggests a potential counterbalancing factor in the early stages of infection, aiming to mitigate the accelerated immune clearance of the capsule-deficient mutant. We postulated that the capsule might influence *A. baumannii* adherence to lung epithelial cells, a crucial step in the infection process in the experimental pneumonia model. Interestingly, we observed significantly greater adherence of the *wza*# mutant to the human lung epithelial cell line A549 compared to the WT or *wza*-Rev strains (Figure 3C). This finding suggests that a defect in lung cell adherence is not responsible for the in vivo attenuation phenotype. Instead, it indicates that a polysaccharide capsule confers protective benefits against immune system clearance at the expense of epithelial adherence capacity. This inverse correlation between hyper encapsulation and epithelial cell adherence potential parallels observations in other Gram-negative human bacterial pathogens such as *Klebsiella pneumoniae* [24] *Escherichia coli* K1 [25].

## 3. Discussion

Understanding *A. baumannii* disease mechanisms is of paramount importance, given its significant impact and rapidly expanding antimicrobial resistance. Recent estimates suggest *A. baumannii* accounts for 1.6% of all healthcare-associated infections in the U.S., with an attributable mortality rate ranging between 21–56% [4,26,27,28]. Despite its clinical relevance, much remains to be elucidated regarding how *A. baumannii* survives in challenging host environments and evades host defenses. A major challenge in *A. baumannii* research has been the need for extremely high inoculums or host immunosuppression to establish a productive infection in mice [29,30,31]. The isolation and characterization of the Lac-4 hypervirulent strain have proven fortuitous, as it infects healthy mice at significantly lower doses than other commonly used strains [17,18].

The Ab Lac-4 genome has recently been sequenced, revealing several unique loci with potential virulence characteristics [19]. Among these loci, the heme oxygenase gene cluster (*hemO*) was identified to contribute to serum and gallium nitrate resistance, iron acquisition, and extrapulmonary dissemination [32]. Another significant finding is the presence of a unique six-gene cluster in the capsule or K locus, likely catalyzing the biosynthesis of α-8-epi-legionaminic acid [19,33], one of the unusual sugars in the trisaccharide repeating unit. Although the success of *A. baumannii* as a pathogen is attributed to multiple factors, the polysaccharide capsule stands out as an important virulence factor in other strains. Given the large size of the K locus and the presence of multiple unique genes [9,19], we therefore examined the contribution of the capsule to the hypervirulence phenotype exhibited by Ab Lac-4.

In our investigation, we observed that the acapsular mutant *wza*# displayed extreme sensitivity to whole blood, complement, and neutrophil-mediated killing. These findings align with prior studies highlighting the role of the *A. baumannii* polysaccharide capsule in defending against complement and the innate immune system [20,22]. Notably, the Lac-4 K locus encompasses several genes encoding enzymes potentially responsible for the production of sugar precursors to α-8-epi-legionaminic acid, a polysaccharide uncommon in bacterial capsules. Legionaminic acid structurally resembles sialic acid present in mammalian cells [34], suggesting that its inclusion among the trisaccharide repeats may be an adaptation to evade immune surveillance by mimicking the host cell surface [35]. Loss and gain of function studies by Talyansky et al. that manipulated the expression of an N-acetyl-β-D-glucosamine sidechain in the capsule different *A. baumannii* strains (HUMC1 and ATCC 17978) confirmed an important contribution to blood survival and virulence in a murine intravenous challenge model [15]. As Lac-4 is the only strain of the relatively rare sequence type 10 (ST10) to have its genome sequence published thus far [19], it remains to be determined whether our findings extend to other ST10 strains. 

In our study, we also noted that the loss of the capsule resulted in a modest increase in susceptibility to certain antibiotics. *A. baumannii* is notorious for its high level of multidrug resistance, reaching up to 80% prevalence among clinical isolates. In several strains with fully sequenced genomes, resistance genes are often concentrated into extensive regions known as resistance islands. For example, the AYE strain was identified to possess an 86 kb-long resistance island containing 45 resistance genes [36]. Similarly, the Ab Lac-4 genome features multiple smaller resistance islands and various insertion and transposon sequences that confer resistance to six out of eight antibiotic classes [19]. Despite the capsule loss, the lack of significant changes in antibiotic sensitivity suggests that the abundance of antibiotic-resistance genes may be adequate to compensate for most antibiotics. 

Our exploration extended to investigating the role of the capsule in murine models of *A. baumannii* sepsis and pneumonia. In alignment with our in vitro findings, the *wza*# mutant strain exhibited severe attenuation in sepsis-induced mortality and demonstrated significantly reduced dissemination from the lungs during pneumonia. These outcomes emphasize the crucial role of the capsule in animal infection models, suggesting its potential as an appealing conjugate vaccine target. Despite the substantial global clinical burden of *A. baumannii*, no vaccine candidates have successfully reached the market. Nevertheless, a successful vaccine would hold great value in safeguarding vulnerable populations, including the elderly, immunocompromised individuals, and those undergoing prolonged antibiotic treatment. The challenge of selecting the optimal capsule antigen(s) has been hindered by the extensive diversity of capsule types and glycan structures across clinically relevant *A. baumannii* strains [1,9,37]. Whole-cell or outer membrane vesicle (OMV) vaccine strategies have encountered challenges owing to the highly immunogenic and toxic nature of lipopolysaccharide (LPS) in *A. baumannii*. Remarkably, *A. baumannii* is one of only three bacteria known to tolerate the loss of LPS. Clinical isolates have been observed to modify the lipid A component, leading to the emergence of LPS-minus strains that are resistant to colistin while retaining their virulence potential [1,38,39]. This finding suggests that LPS-minus strains could potentially serve as a basis for addressing some of the challenges associated with antigen selection in vaccine development.

The polysaccharide capsule plays a pivotal role in a multifaceted strategy that enables *A. baumannii* to thrive in highly unfavorable built environments, making it a significant contributor to nosocomial infections. This study outlines a specific contribution of the capsule in the hypervirulent clinical isolate *A. baumannii* Lac-4, highlighting its critical function as a defense against the innate immune system and its essential role in in vivo pathogenicity.

## 4. Materials and Methods

### 4.1. Bacterial Strains and Culture

The *A. baumannii* strain Lac-4 was obtained from Wangxue Chen (National Research Council, Ottowa, ON, Canada). Lac-4 and mutant strains described below were grown in tryptic soy broth (TSB) with aeration at 37 °C. Stationary phase overnight cultures were sub-cultured in TSB and grown to mid-logarithmic phase with aeration at 37 °C and washed with PBS prior to use in experiments. *Escherichia coli* DH5α was grown at 37 °C with aeration in LB, supplemented with 15 μg/mL apramycin where indicated. Electrocompetent cells were prepared by growing *E. coli* DH5α and *A. baumannii* Lac-4 to OD600 ~0.4 in LB or TSB, respectively, washing three times in ice-cold dH_2_O, and resuspending the final pellet in dH_2_O + 10% glycerol. Electrocompetent cells were snap-frozen and stored at −80 °C.

### 4.2. Creation of A. baumannii Mutant wza#

The apramycin resistance gene was amplified (Table 1) from pKO (Apr) (Addgene, Watertown, MA, USA, #110088) and inserted into pMo130 (Addgene, #27388) using Gibson Assembly (NEB, Ipswich, MA, USA, #E2611S) and electroporated into *E. coli* DH5α using an Eppendorf electroporator 2510 at 1600 mV. Electrocompetent cells were recovered in SOC media for 90 min at 37 °C with aeration and plated on LB + 15 μg/mL apramycin. The successful transformation was confirmed by plasmid purification (Qiagen, Hilden, Germany, #27104) and sequencing. To generate the knockout vector pMo130Apr-*wza*UpDown, genomic DNA was isolated from *A. baumannii* Lac-4 using a DNeasy Blood and Tissue kit (Qiagen, #69504). Regions of ~0.5 kb both upstream and downstream of *wza* were amplified from *A. baumannii* Lac-4 gDNA with overlapping restriction enzyme sites. pMo130Apr and the upstream product were double digested with NotI-HF and BamHI-HF (NEB, #R3189, #R3136). Digests were run on a 1% agarose gel, and appropriate bands were excised and purified using a QIAquick gel extraction kit (Qiagen, #28706). The vector and insert were ligated using ElectroLigase (NEB, #M0369S) and electroporated into DH5α as described above. The successful transformation was confirmed using sequencing. pMo130Apr-*wza*Up and the *A. baumannii* Lac-4 downstream PCR product were double digested with BamHI-HF and SphI-HF (NEB, #R3182). Digests were run on a 1% agarose gel, and appropriate bands were excised and purified using a QIAquick gel extraction kit. The vector and insert were ligated using ElectroLigase and electroporated into DH5α as described above. The successful transformation was confirmed using sequencing. For single-crossover selection, pMo130Apr-*wza*UpDown was electroporated into electrocompetent *A. baumannii* Lac-4 cells at 2500 mV, recovered in SOC media, and plated on LB + 50 ug/mL apramycin. Single-crossover mutants were confirmed using PCR disruption of the upstream or downstream regions. For double-crossover selection, mutants were counterselected by serial passaging in 20% sucrose for 72 h before serially diluting and plating onto 2xYT + 20% sucrose. Double mutants were screened by loss of apramycin resistance and decreased buoyancy, then confirmed using DNA sequencing. *wza*-Rev was selected from mutants that lost apramycin resistance while maintaining buoyancy and mucoid appearance. Primers used in mutant construction are found in Table S1. 

### 4.3. Minimum Inhibitory Concentration (MIC) Testing

The MIC_90_ for colistin (Sigma, St. Louis, MO, USA, #C4461), polymyxin B (Sigma, #P0972), penicillin (Sigma, #5161), streptomycin (Sigma, #S9137), ampicillin (Sigma, #171254), vancomycin (Sigma, #SBR00001), doxycycline (Sigma, #D1822), cefepime (Sigma, #Y0000633), meropenem (Sandoz, Basel, Switzerland, #0781-3000-94), piperacillin/tazobactam (Sigma, #93129, #T2820), imipenem (Sigma, #I0090000), and levofloxacin (Sigma, #40922) was determined for *wza*# and *wza*-Rev. Briefly, *A. baumannii* strains were grown to OD600 of ~0.4, washed twice with PBS, resuspended at an OD_600_ of 0.4 (~1 × 10^8^ CFU/mL), and diluted 1:100 in RPMI supplemented with 10% TSB. Antibiotics were serially diluted two-fold from 250 μg/mL to 1.95 μg/mL. Bacteria were added to the diluted antibiotics and grown for 20 h. MIC_90_ was determined as the concentration at which inhibited 90% or more growth relative to untreated.

### 4.4. Whole Blood and Serum Killing Assays

WT, *wza*#, and *wza*-Rev were incubated with whole blood or 10% human serum. Briefly, blood was isolated from consenting human donors in accordance with approved protocols UCSD Institutional Review Board, #131002. Whole blood was collected from healthy human donors using venipuncture into BD Vacutainer Plasma Tubes spray-coated with sodium heparin (BD, Franklin Lakes, NJ, USA, 367874). Serum was isolated from blood collected using venipuncture into BD Vacutainer Serum Separator Tubes (BD, 367985) from three male and three female donors. SST tubes were spun at 2500 RPM for 15 min, and the serum was pooled. Whole blood and serum were heat-inactivated at 56 °C for 30 min.

### 4.5. Opsonophagocytic Killing Assays

WT, *wza*#, and *wza*-Rev *A. baumannii* were incubated with healthy human neutrophils as previously described [40]. Briefly, whole blood was collected into sodium heparin vacutainers (BD, 367874) using venipuncture from human donors as above. Whole blood was layered onto PolymorphPrep (Proteogenix, Miami, FL, USA, 11146832) and spun, and the neutrophil layer was isolated. Neutrophils were washed, and red blood cells were lysed. Bacterial strains were incubated with 10% heat-inactivated human serum for 30 min at 37 °C. Neutrophils were isolated from consenting human donors under approved protocols (UCSD Institutional Review Board, #131002) and incubated at a multiplicity of infection (MOI) of 1 bacterium to each neutrophil for 3 h at 37 °C with 5% CO_2_. Bacterial was assessed using lysing neutrophils with dH_2_O, serially diluting in sterile PBS, and plating on TSA (Sigma, 22092).

### 4.6. Adhesion Assays

A549s were obtained from ATCC (#CCL-185) and grown in tissue culture flasks (Corning, Corning, NY, USA, #430825) in RPMI (Gibco, Waltham, MA, USA, #11873-903) supplemented with 10% FBS (Avantor, Radnor Township, PA, USA, #97068-085). Upon reaching ~80% confluency, cells were washed 2× with room temperature PBS (Fisher, Waltham, MA, USA, #21-031-CV) and lifted with 0.25% trypsin (Corning, #24-053-CI). 4 × 10^5^ cells/well were seeded into a 96-well TC-treated plate and allowed to adhere overnight. 2 × 10^5^ CFUs log-phase WT, *wza*#, and *wza*-Rev were added to each well and incubated for 30 min. Wells were washed 5× with 200 μL sterile PBS to remove non-adherent bacteria. Cells were trypsinized, serially diluted, and plated on TSA (Sigma, 22092).

### 4.7. Murine Studies

Murine experiments were performed in accordance with the University of California San Diego Institutional Animal Care and Use Committee (IACUC, Protocol #S00227M) and the Animal Welfare Act. All protocols were approved by IACUC prior to experimentation. Male and female mice were housed in pre-bedded corn cob disposable cages (Innovive, San Diego, CA, USA, #M-BTM-C4), fed Teklad global soy protein-free extruded rodent diet (Inotiv, Indianapolis, IA, USA, #2020X), and received acidified water. Mice were housed in a specific pathogen-free (SPF) facility on a 12 h light/dark cycle. Mice were randomized into cages by vivarium staff 3–7 days prior to experimentation. 

### 4.8. Murine Sepsis and Pneumonia

*Sepsis.* 8 to 12-week-old male and female C57BL/6J mice (Jackson Laboratory, Bar Harbor, ME, USA, 00664) were intraperitoneally infected with 10^7^ CFUs log-phase Ab Lac-4, *wza*#, or *wza*-Rev in sterile PBS to induce disseminated sepsis. Mice were monitored twice daily for 7 days. *Pneumonia*. Mice were infected intratracheally with 10^7^ CFUs log-phase *A. baumannii* Lac-4 WT, *wza*#, or *wza*-Rev in sterile PBS. Prior to infection, mice were anesthetized with 100 mg/mL ketamine and 10 mg/mL xylazine. Mice were monitored on a heating pad until they fully recovered from anesthesia. Twelve h post infection, mice were humanely euthanized with CO_2_ and confirmed using cervical dislocation according to IACUC-approved protocols. Blood was obtained using cardiac puncture. The spleen and lungs were collected for bacterial burden assessment. Organs were disrupted with a mini Beadbeater-96 (BioSpec, Bartlesville, OK, USA, #1001) for 2 min at 2400 rpm, serially diluted, and plated for CFU enumeration.

### 4.9. Statistical Analysis and Programs

Statistical analysis was performed using GraphPad Prism v8. One-way analysis of variance (ANOVA) with pairwise comparisons and Bonferroni post-test correction were used to compare significance across multiple groups. Matched samples were compared with two-way ANOVA with Tukey’s multiple comparisons test. Log-Rank (Mantel-Cox) tests were used for the analysis of survival. Values of *p* < 0.05 were considered statistically significant. 

## Figures and Tables

**Table 1:** Polysaccharide capsule contribution to antibiotic susceptibility. Minimum inhibitory concentrations (MIC90) for Ab Lac-4, *wza*#, and *wza*-Rev grown in the presence of various FDA-approved antibiotics. Means of three to five independent experiments ± SEM.

	*wza*#			*wza*-Rev		
	Mean	SEM	*p*	Mean	SEM	*p*
**Doxycycline**	1.75	0.75	n.s	1.37	0.22	n.s
**Cefapime**	1.15	0.30	n.s	1.65	0.24	n.s
**Colistin**	1.00	0.00	n.s	1.00	0.00	n.s
**Polymyxin B**	1.00	0.00	n.s	1.00	0.00	n.s
**Ampicillin**	0.86	0.14	n.s	0.71	0.29	n.s
**Meropenem**	0.88	0.15	n.s	0.97	0.11	n.s
**Penicillin**	0.89	0.06	n.s	0.78	0.15	n.s
**Imipenem**	0.67	0.17	n.s	3.00	1.53	n.s.
**Streptomycin**	0.56	0.06	n.s	1.00	0.00	n.s
**Vancomycin**	0.33	0.04	n.s	0.78	0.31	n.s
**Piperacillin/Tazobactam**	0.43	0.10	n.s	0.86	0.21	n.s
**Levofloxacin**	0.25	0.00	n.s	2.00	0.00	n.s

## Data Availability

Data generated or analyzed during this study are included in this published article.

## Supplementary Materials

The following supporting information can be downloaded at: https://www.mdpi.com/article/10.3390/antibiotics13010010/s1, Figure S1: Targeted mutation confirmation; Table S1: Primers used in mutant construction.
