# Supplementary material for: Exploring Roles of the Polysaccharide Capsule in Pathogenesis of Hypervirulent Acinetobacter baumannii Clinical Isolate Lac-4
Source: Antibiotics (Basel). 2023 Dec 20;13(1):10. doi: 10.3390/antibiotics13010010 (PMC10812722; doi:10.3390/antibiotics13010010)
Supplement: Supplementary file 1 [file antibiotics-13-00010-s001.zip › antibiotics-2769904-supplementary.pdf]

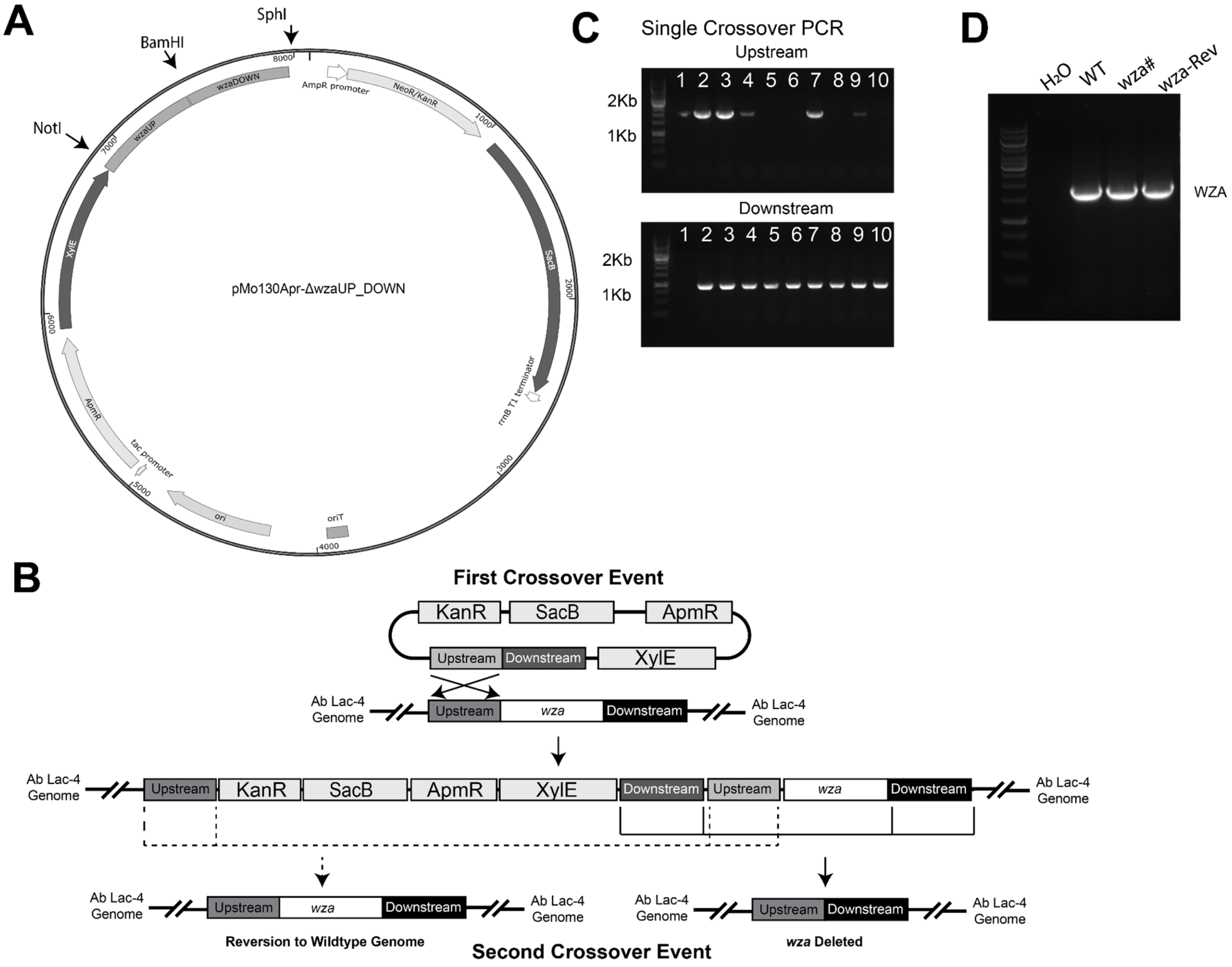

Figure S1: Targeted mutation confirmation. (A) Plasmid map for pMo130AprΔwza-UpDown containing Apramycin resistance gene, *SacB* for sucrose selection, and *wza* upstream and downstream flanking regions. B) *wza#* was generated using a two-step selection process. Single crossover insertional mutants containing the suicide vector pMo130Apr and complementary upstream and downstream regions were selected for using apramycin resistance. Double crossover deletion mutants were selected with serial passaging in sucrose. A wildtype revertant (*wza*-Rev) was chosen as a control. Mutants were confirmed by PCR and sequencing. C) Confirmation of successful single-crossover insertional mutants by PCR showing disruption of upstream region. Lanes 1) H_2O, 2) Ab Lac-4, 3-10, single crossover clones 1-8. D) PCR showing *wza* gene still present in *wza#*. Lanes 1) H_2O 2) Ab Lac-4 3) *wza#* 4) *wza*-Rev.

Primer Name	Primer sequence
Apr_Forward:	5′-agtgccgttgatcgtgcta-3′
Apr_Reverse	5′-cctccaacgtcatctcgttctc-3′
pMo130_Forward	5′-tcgccccttttaattgagaaggtgatcgactgatgtcat-3
pMo130_Reverse	5′-gctcctgtcgcaattaccgcgtgatattccacacattatacgagcc-3′
wza_Up_NotI_Forward	5′-GTACTCTAGGCGGCCGCgttccagcagctttaagcacatc-3′
wza_Up_BamHI_Reverse	5′-GTATGCTAGTAGGATCCcacaagaattacaacaagcggtgca-3′
wza_Down_BamHI_Forward	5′-CTAGTATCTAGGATCCacagtggaaattaattgcactctgcac-3′
wza_Down_SphI_Reverse	5′-GTACTAGTACGCATGCtgttcttttgtaaagatggcgactttccata-3′

Table S1: Primers used in mutant construction.
